# Mental Health Issues as a Consequence of the Pandemic: Group Psycho-Educational Intervention to Support Patients Recovered from COVID-19 Infection

**DOI:** 10.3390/ijerph20126105

**Published:** 2023-06-12

**Authors:** Denise Vagnini, Nicolò Lamperti, Sara Molgora, Francesca Barile, Federica Facchin, Umberto Mazza, Emanuela Saita

**Affiliations:** 1 Department of Psychology, Università Cattolica del Sacro Cuore, 20123 Milan, Italy; 2 Clinical Psychology Unit, Department of Mental Health and Addiction Services, ASST Grande Ospedale Metropolitano Niguarda, 20162 Milan, Italy

**Keywords:** COVID-19, group intervention, psycho-education, cognitive-behavioral approach, mental health, hospitalized patients

## Abstract

Common psycho-physical symptoms have emerged in patients who were previously recovered for COVID-19 infection, including traumatic experience and enduring emotional disturbances. A group psycho-educational intervention of seven weekly sessions and a follow-up after three months was proposed to all Italian-speaking patients formally discharged from a public hospital in northern Italy and physically recovered from infection. Eighteen patients were recruited and divided into four age-homogenous groups, each led by two facilitators (psychologists and psychotherapists). The group sessions followed a structured format with thematic modules, including main topics, tasks, and homework assignments. Data were collected through recordings and verbatim transcripts. The objectives of the study were twofold: (1) to analyze the emerging themes and gain insight into the significant aspects of the participants’ lived experience of COVID-19, and (2) to examine changes in how participants approached these themes throughout the intervention process. Semantic-pragmatic text analyses, specifically thematic analysis of elementary context and correspondence analysis, were conducted using T-LAB software. Linguistic analysis revealed a congruence between the intervention’s objectives and the participants’ experiences. The study highlighted an evolution in the narratives, as participants transitioned from a passive and concrete perspective on the disease to a more comprehensive cognitive and emotional elaboration of their personal illness stories. These findings hold potential relevance for healthcare services and professionals working in this field.

## 1. Introduction 

### 1.1. COVID-19 and Mental Health

The COVID-19 pandemic had a tremendous impact on society from multiple perspectives, and led to increased medical, socioeconomic, and interpersonal uncertainties [1]. The negative psychological consequences of the pandemic on the general population have been highlighted in numerous studies [1,2,3]. At the same time, the pandemic posed great challenges to the provision of mental health services [4].

Italy was one of the western countries that was most affected by COVID-19, at least in the initial phase of the pandemic, with a huge number of cases reported, especially in northern Italy. At that time, there were significant regional differences between the north and south of Italy; Lombardy was the worst-hit region [5], and the psychological impact of that situation on the people was reported in several Italian studies [6,7,8,9]. 

Impaired psychological health was associated with the emotional burden of the pandemic [10], which also involved containment measures applied by the governments, along with the exposure of people to feelings of uncertainty, fear, and anger, not to mention the economic crisis [11]. Specifically, a huge body of research showed people’s higher levels of anxiety and depressive symptoms, distress, and posttraumatic symptoms compared with the pre-pandemic period [12]. Furthermore, those symptoms were associated with several physical problems such as sleeping disorders and worse eating habits [2]. Although some of these symptoms decreased over time, other mental health problems remained unchanged or even increased, which clearly indicates the need for implementing treatment and prevention programs [13]. 

The negative psychological impact of the pandemic is even more evident among people who directly experienced COVID-19 infection. Being infected with the virus entails being quarantined and consequently isolated for an extended period, which further adds to the stress experienced [9]. In this regard, the presence of posttraumatic symptoms among people diagnosed with COVID-19 was highlighted in several studies, along with a variety of negative psychological outcomes such as symptoms of anxiety and depression, also related to the long-term consequences of the disease [14,15]. On October 2021, the World Health Organization (WHO) defined the “Post-COVID-19” experience as a “condition that occurs in individuals with a history of probable or confirmed SARS-CoV-2 infection, usually 3 months from the onset of COVID-19 with symptoms that last for at least 2 months and cannot be explained by an alternative diagnosis […]” [16] (p. 11). Therefore, patients could experience a multi-systemic syndrome that has an impact on everyday functioning and entails a protracted presence of multiple physical symptoms, such as fatigue and weakness, with the onset or persistence of psychological symptoms such as anxiety, depression, posttraumatic stress disorder (PTSD), obsessive-compulsive symptoms, cognitive impairments (i.e., brain fog), and delirium in older adults. These physical and psychological symptoms may be present from the onset of the disease, or fluctuate, with little relationship between previous illness severity and people’s likelihood of developing a protracted post-COVID-19 symptomatology [16,17,18,19,20,21].

The probability of developing long-term symptoms as a consequence of contagion is substantial. Significant long-term consequences of the disease were reported by COVID-19 survivors in terms of PTSD, anxiety, depression, and psychological distress, with percentages ranging from 20% of individuals for PTSD to 36% of individuals for psychological distress [22]. Due to the post-COVID-19 syndrome, patients may be unable to cope with daily life, especially if they also experience significant loneliness, stigmatization, and are unable to return to work for long periods [23]. 

A recent meta-analysis [24] estimated that about 80% of the people infected with the virus experienced one or more long-term symptoms, especially fatigue. Similarly, in another Italian study [25], about 87.4% of discharged patients reported a persistence for a year of at least one symptom, and these data are consistent with a meta-analysis that examined the global prevalence of the disease [26]. In this scenario, social support has been identified as the strongest protective factor, and people at risk (e.g., discharged patients) should receive different forms of support, provided by either formal (e.g., targeted interventions offered by healthcare services) or informal networks [27,28]. 

Overall, the health effects of post-COVID-19 could impact people’s quality of life and cause mental health issues, as well as put additional burden on healthcare systems. These patients require psychological support and multidisciplinary rehabilitation, and resilient and efficient healthcare systems are needed to ensure prompt responses through specific intervention protocols, in order to be able to deal with future health challenges [23,26,29].

In this context, preliminary research on interventions for people following recovery, or at risk of burnout (e.g., healthcare workers) has grown rapidly, especially regarding e-health programs. For example, Fan et al. [30] studied the effects of a Narrative Exposure Therapy and a personalized psychological intervention in China, highlighting a positive effect on the reduction of posttraumatic stress symptoms. In Europe, a study conducted in Germany by Bäuerle et al. [31] evaluated the effectiveness of a self-guided e-mental health intervention composed of modules with psycho-educational videos, mindfulness practices, and short trainings to strengthen adaptive skills (e.g., stress management) in the general population. This intervention seemed to be effective in reducing distress, anxious and depressive symptoms, and in enhancing perceived self-efficacy. In France, Bureau et al. [32] evaluated the feasibility of an internet-based CBT intervention for healthcare workers aimed at increasing resilience to stress and preventing mental health problems through digital easy-to-use resources based on psycho-educational, mindful and breathing techniques. The study highlighted positive results consistent with the scientific literature on online self-guided mindfulness-based interventions and CBT. Similarly, in the Italian context, Bossi et al. [33] tested the effectiveness of an online mindfulness intervention on healthy employees and found a positive effect on nonreactivity, positive affect, depression, and insomnia. Instead, Rossi Ferrario et al. [34] proposed a structured psychological support intervention based on a CBT framework to hospitalized post-acute COVID-19 patients, showing that many of them reported psychological issues, along with the need for psychological rehabilitation. However, there is poor research literature regarding these types of interventions in the Italian context (especially the most hit regions, such as Lombardy, during the first wave). 

### 1.2. Group Psycho-Educational Intervention for Patients Recovered from COVID-19 Infection: Where We Started and the Structure of the Intervention

In 2020, the Clinical Psychology Service of a large hospital located in northern Italy participated in a surveillance and intervention project promoted by the hospital and aimed at supporting psychological health in patients affected by COVID-19. The project was multidisciplinary and comprised clinical, medical, and psychological evaluations of all patients discharged after one or more admissions for COVID-19 infection. It involved (from June 2020 to November 2022) a total of 1208 patients, who were evaluated by diagnostic screening to detect early signs of psychological distress, especially focusing on prodromic clinical symptoms of adaptation disorders or posttraumatic stress. Each patient was then evaluated at the 12-month follow-up.

The clinical assessments revealed the presence of remarkable psychological difficulties in terms of regulation of negative emotions and poor functional adaptation to the resumption of daily life. Specifically, all patients who showed poorer psychological conditions at the preliminary assessment were taken in charge and treated with individual interventions, both remotely and in presence. 

The hospital was able to offer on average eight individual psychological consultations with a specialist (i.e., a certified psychologist psychotherapist) of the Service to 532 (44%) patients. The limited number of psychological consultations was due to the health emergency and undersize of the available staff compared to the demand for help. It is important to note that these 532 people were the new patients taken in charge by the specialists of the Service that were to be added to the patients already in charge before the pandemic.

At the end of these psychological consultations, some patients still presented a critical clinical state. These patients were evaluated by the psychologists who had them in charge considering different criteria: levels of anxiety and depression, PTSD symptoms, duration of the hospitalization, loss of close ones due to COVID-19 (especially in case of complicated grief), and difficulty in re-entering the working environment. 

As a result of this evaluation, a group intervention was proposed to support these patients. This intervention was designed with a modular psycho-educational group structure, aimed at promoting adaptive and functional coping strategies, and restoring or improving people’s sense of self-efficacy [28,35].

The psycho-educational group intervention for COVID-19 patients mentioned above, divided into thematic modules, was originally developed by Weiner et al. [36]. The original version of this intervention was delivered online using video-conferencing platforms. A similar modular approach was also used with COVID-19 patients by Bryant et al. [37] to implement an individual intervention. The efficacy of such a group intervention has been highlighted by the literature [38].

The structure of our intervention was also outlined considering the intervention model developed by Wakefield et al. [39] and offered to the family of young people with cancer. We also considered that the extant research identified cognitive-behavioral approaches and brief awareness-building interventions as the most effective evidence-based techniques with patients in the post-COVID-19 period [40,41]. 

In our program, it was deliberately opted for conducting face-to-face groups, using dedicated spaces within the hospital, in order to: (1) rebuild participants’ awareness of the possibility of social support, despite the containment measures and the limitations caused by seeing the other as a potential source of contagion [42]; (2) bring the patients back to the place where they developed their traumatic experiences, building a connection that repositions the hospital as a place of care and promotion of well-being. The choice of a group setting also responded to the need for a cost-effectiveness ratio, considering the number of patients and the length of treatment. The intervention was structured in seven sequential sessions, on a weekly basis, with an additional follow-up meeting (session eight) held after three months.

The groups were organized as closed groups, ensuring homogeneity in terms of participants’ age. The sessions were facilitated by two psychotherapists or psychologists who received specific training and were part of the Clinical Psychology Service at the hospital. 

Due to the face-to-face in-presence modality, national guidelines (e.g., the use of personal protective equipment) had to be maintained as measures of protection for public health during a health emergency. In addition, the group intervention was designed taking into account the guidelines received to ensure interpersonal distance in spaces with adequate ventilation systems without recycling. Considering the characteristics of the available spaces at the hospital, it was possible to form groups with a maximum of eight participants each, including operators (i.e., the conductors).

The intervention design was organized in thematic modules, each focused on a main topic and followed by classroom exercises and homework on the addressed topic. The materials used in the meetings were derived from the work of Ruggiero and Sassaroli [43], Leahy et al. [44], and Segal et al. [45]. A more comprehensive description of the intervention is provided in Table 1. The sessions were audiotaped with participants’ consent. 

## 2. The Current Study: Aims

In the current exploratory study, we performed an analysis of the language used by the participants during the scheduled group sessions. Specifically, we conducted a semantic-pragmatic computer-assisted analysis of the intervention process using a qualitative approach and starting from the spoken language (derived from verbatim transcriptions) of the participants. 

According to Carli and Paniccia [50], in order to understand the surrounding reality, people need to give it an emotional meaning, which is later shared with others living in the same context; in this process, spoken and expressed language organizes relationships both among individuals and with their living environment. In addition to this theoretical framework, the current study was based on the idea that the analysis of language allows for exploring the cultural models and representations shared by the people who live in the same context and experience a similar situation, such as being infected by COVID-19 and participating in the same group intervention program. In other words, language conveys deeper symbolic and emotional meanings characterizing human experience. 

The objectives of this study were:

(1) to analyze the emerging themes during the sessions, including their frequency, in order to describe and understand the most significant aspects of the participants’ lived experience of COVID-19;

(2) to examine any changes in how participants addressed these themes throughout the intervention, specifically observing any differences in their language usage between the first and eighth sessions. While this study is exploratory in nature, we hypothesized that the group psycho-educational intervention process would promote an enhanced emotional-oriented elaboration of participants’ illness experiences by facilitating the expression of their thoughts and emotions within a group setting.

## 3. Materials and Methods

### 3.1. Participants and Data Collection Procedure

This study involved group interventions conducted with four separate and closed age-homogenous groups. Each group consisted of a maximum of five participants and was facilitated by two operators, specifically psychologists and psychotherapists, with an expertise in conducting group therapies and aftercare. This study included a total of 18 participants who were patients aged > 18 years, had experienced hospitalization due to COVID-19 infection, and were fluent in Italian. All the participants were no longer hospitalized when the intervention was conducted; specifically, the time between hospital discharge and the participation at the group intervention varied from six to nine months.

The intervention was delivered face-to-face in a dedicated room within the hospital, which ensured privacy and adequate interpersonal distance between participants (because of restrictions to limit contagion and protect health). The length of each session was approximately two hours. The seven weekly sessions were conducted from April to June 2021, and the follow-up session was held in September 2021. 

This study was conducted retrospectively from data obtained for clinical purposes, and all the procedures performed were part of the surveillance and intervention project promoted by the hospital to support patients infected with COVID-19. Data were accurately anonymized from clinicians and subsequently used for research purposes. For this reason, ethical approval was formally waived by the Ethics Commission of the Department of Psychology at Università Cattolica del Sacro Cuore, Milan, Italy (CERPS: Commissione Etica per la Ricerca in Psicologia), protocol N° 01-23 (10/02/2023). Participants were informed about the objectives and procedures of the intervention; they were also informed that the material collected during the intervention could be anonymized and used for scientific purposes. They also were required to give informed consent for audiotaping. 

The audiotapes were then transcribed verbatim in Italian; therefore, the quotes/words reported in this article were translated into English by the authors. Any identifying detail was removed from the transcripts and participants’ names were replaced with progressive ID numbers to protect their anonymity. Qualitative analyses were conducted on these anonymized verbatim transcriptions of the group sessions.

General participant characteristics (i.e., age, gender, work status, prescribed therapy for COVID-19 infection, days of hospitalization, and mourning of a beloved one due to COVID-19) were collected using an anonymous self-report data sheet with open questions, completed by the participants before the first group session.

Finally, before the first and after the final group session, the procedure also included the administration of psychometric tests scientifically validated for the assessment of patients’ psychological health (i.e., symptoms of anxiety and depression, coping strategies, emotion regulation, and self-efficacy). However, in this contribution, we focused only on the qualitative analysis of the intervention process. 

### 3.2. Data Analysis

Descriptive statistics on general characteristics of the sample (i.e., age, days of hospitalization, gender, work status, prescribed therapy for COVID-19 infection, mourning of a beloved one due to COVID-19) were derived using IBM SPSS Statistics 27. 

Then, we performed linguistic analysis using the word-driven and question-oriented textual analysis software T-LAB, version 9.1.2 (Italy) [51]. It is a Computer-Assisted Qualitative Data Analysis Software (i.e., C.A.Q.D.A.S.) based on a mixed method approach (i.e., quantitative and qualitative), that through numerous algorithms allows us to conduct a series of in-depth exploratory and interpretative text analysis, using a set of linguistic, statistical, and graphical tools. The software generates new data and allows the analysis and comparison of the lexical characteristics of a text (named corpus in T-LAB specific language). T-LAB calculates how often a single word (referred to as lexical unit) appears in the whole text (i.e., corpus) or in parts of it. This type of analysis was named “Occurrences”. Moreover, T-LAB calculates how many times two or more lexical units appear together in the same part of the text (i.e., analysis of the “Co-occurrences”). The corpus can be divided by the researcher into smaller parts identified by encoding strings. Overall, through specific statistical indexes and *p*-values for significance, T-LAB supports the researcher in the evaluation of the lexical units, in the exploration of the semantic specificities of the text, and in the comparison of these specificities between different parts of the texts. 

#### 3.2.1. Corpus Preparation and Text’s Encoding Strings

In this study, the encoding strings identified the eight sessions. Therefore, the corpus was divided into eight parts using the string **** *SESSION_n (from **** *SESSION_1 to **** *SESSION_8). The corpus was “cleaned” to: (1) eliminate ambiguities (e.g., in case of homographs, words with the same root but with a different meaning); (2) group synonymous under the same label, and (3) recode and anonymize personal names (along with any identifying detail). In its final form, the corpus comprised 502 words that were included in the analyses with an occurrence threshold value of 10 or more.

#### 3.2.2. Thematic Analysis of Elementary Contexts

The main themes addressed by the participants (aim 1) were studied using an “exploratory” approach to the text with a procedure named “Thematic Analysis of Elementary Context”. This analysis allowed for the identification of thematic clusters (i.e., patterns of words/lexical units that are more likely to appear together within the corpus) derived from the analysis of the co-occurrences between words. The clusters are generated using a maximum similarity criterion: the lexical units within the same cluster are maximally similar, and maximally different from the words that characterize the other clusters. In this way, according to Lancia [51], each cluster allows the reconstruction of “a thread” of the discourse within the overall plot of the corpus.

The results consisted of a list of lexical units occurring within each cluster and associated with a χ^2^ value and a *p*-value for significance (this means that the statistical analysis on which it is based is χ^2^), and significant portions of text characterized by the same pattern of words/lexical units. A graphical representation of the clusters showed their placement in the factorial space. The automatic and “unsupervised” clustering method (bisecting K-Means), included within the functions of T-LAB, was chosen to conduct the analysis. Using this procedure, thematic clusters were generated by T-LAB, and each cluster was given a label (i.e., a “title”) by the researchers, according to the words (with high χ^2^ value and significant *p*-value) characterizing the cluster. 

The graphical tools integrated in T-LAB were then used to examine the frequency of each theme in the eight sessions. In this case, the output was a histogram with the percentage presence in the eight group sessions of each thematic cluster. 

#### 3.2.3. Correspondence Analysis

The Correspondence Analysis is a comparative analysis that allows for highlighting relations of similarity and difference from a linguistic point of view between parts of the corpus. In our study, we used this analysis to examine similarities and differences between sessions with regards to the themes identified in the previous analysis (aim 2). These relations are displayed in tables and graphs, and the statistical logic of this analysis is factorial analysis. 

The output extracted: (1) new spatial vectors, called factors (latent dimensions) with a positive and a negative pole, which were informative of the relationship between the eight group sessions, and (2) a graphical representation of the distribution of these factors in the factorial space, and the location of each group session. The graph was centered at the value 0, the middle point between the negative and positive factorial poles. The group sessions close to the factorial poles are considered more different from each other.

To interpret these results, we examined the test values (i.e., the saturation values ≥ |1.96|) and the associated *p*-values (*p* ≤ 0.05) of the lexical units at the positive and the negative pole of each factor, considering that higher significant values characterized more important elements in saturating the factor pole. Then, we studied the difference between the lexical units opposed to the factorial poles, also giving a label to the vertical and horizontal vectors.

## 4. Results

### 4.1. Descriptive Statistics

Participants were 18 patients, homogenous in terms of socio-demographic characteristics, and with a mean age of 62.68 ± 8.19 years. Ten patients received only pharmacological treatment for COVID-19, five received Continuous Positive Airway Pressure (CPAP) ventilation therapy, and three needed hospitalization in the intensive care unit. On average, patients spent 25.28 ± 22.89 (min = 6; max = 84) days hospitalized due to COVID-19 infection. Participants’ characteristics are summarized in Table 2. 

### 4.2. Thematic Analysis of Elementary Contexts

**Figure 1:**
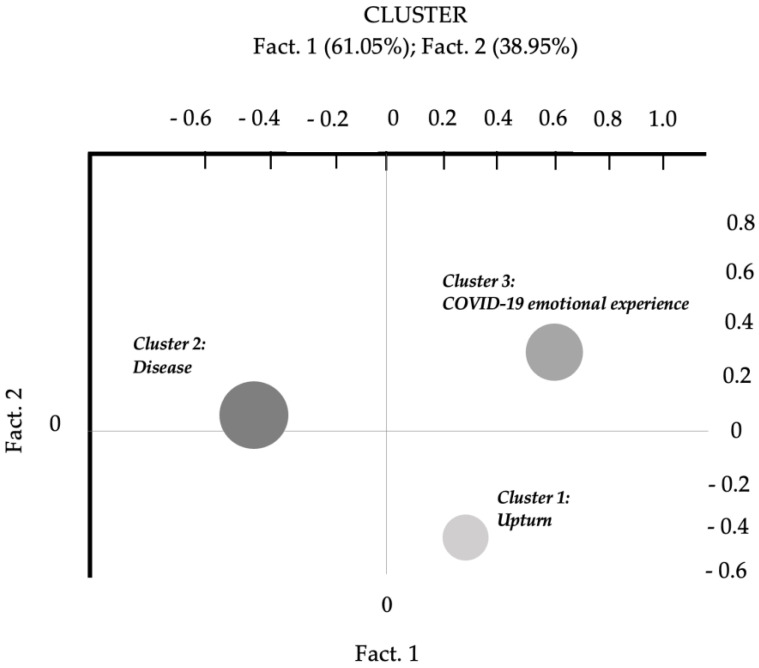
Thematic Analysis of Elementary Contexts: distribution of the three-cluster solution in the factorial space.

Thematic Analysis of Elementary Contexts was performed on the whole corpus to achieve our first research aim. The final output showed a three-cluster solution that highlighted the main thematic cores of participants’ experience (Figure 1).

Clusters were studied in order of explained variance: Cluster 2 = 48.40%; Cluster 3 = 26.45%; and Cluster 1 = 25.15%. 

Cluster 2 included words (i.e., lexical units) such as “doctor”, “COVID-19”, “hospital”, “to hospitalize”, “to miss”, and “pain”, which indicate contextual aspects related to the COVID-19 infection, places related to the disease experience, and participants’ experience of physical pain. The language used by the participants reflects a very concrete, descriptive, and detailed story of the experience of COVID-19. For this reason, Cluster 2 was entitled “Disease”.

Cluster 3 reflected other aspects of the illness, such as the emotional experiences related to the disease and the overall pandemic situation, and the perception of being in deadlock, unable to move forward. Participants’ language highlights an emphasis on the feelings experienced, with lexical units such as “anxiety”, “to think”, “fear”, “nightmare”, and “anger”. This cluster was labelled “COVID-19 emotional experience”, to differentiate the emotional, subjective dimension from the factual and more objective story reflected by Cluster 2. 

Cluster 1 was characterized by a positive and evolutionary perspective of change, with lexical units such as “power”, “strength”, “life”, “to move on”, “to take up again”, and “motivation”. That is why Cluster 1 was entitled “Upturn”, with the aim of placing emphasis on the aspects of rebirth and self-confidence for the future. Table 3 summarizes the characteristics of the three clusters, with meaningful lexical units and significant χ^2^ values, and contains exemplificative quotes from participants’ voices. 

The positioning of the three clusters in the factorial space suggests the dynamic intersection of two dimensions, explaining 61.05% (Fact. 1) and 38.95% (Fact. 2) of the variance, respectively. 

Factor 1, the vertical vector, compared Cluster 3 (“COVID-19 emotional experience”) and Cluster 1 (“Upturn”) and was entitled “Attitude and coping style”, and interpreted as a continuum from a condition of “Rumination and helplessness” to “Future orientation and mastery of events”. This continuum underlines different abilities to activate oneself and move with respect to the feelings of being in a deadlock and distress experienced during COVID-19. Factor 2, the horizontal axis, seems to describe the transition from a more factual narrative about the disease and the hospitalization (see Cluster 2, “Disease”, oriented towards the negative pole) to a greater capacity of reflexivity and desire to share emotions and feelings with the others in the group (Cluster 1 and Cluster 3).

**Figure 2:**
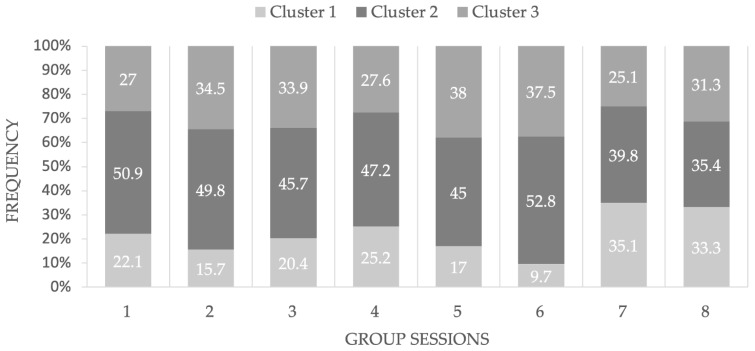
Presence in percentage of the extracted thematic clusters* by group session. * Cluster 1 (“Upturn”) in the light gray; Cluster 2 (“Disease”) in the darker gray; Cluster 3 (“COVID-19 Emotional Experience”) in gray.

Finally, the analyses carried out to explore the frequency of each theme (i.e., cluster) in the eight group sessions revealed that Cluster 1, “Upturn”, was more likely to occur in the final sessions (35.1% in session_7, and 33.3% in session_8) compared with the initial sessions, which may reflect the positive effects of the group intervention. Figure 2 presents these findings.

### 4.3. Correspondence Analysis

Correspondence Analysis, performed to examine similarities and differences between sessions, led to the extraction of some new factors. Specifically, the number of new factors is defined by a software algorithm, according to this formula: number of factors = number of levels of the design variable considered − 1. 

In the current study, the design variable was “group session” with eight levels, so the formula to extract the number of factors was: 8 − 1 = 7.

The authors chose to interpret the first two factors because they explain a relevant percentage of the variance. Precisely, Fact. 1 explains 30.01% and Fact. 2 the 14.73% of variance, the largest portion compared to variance explained by all the other dimensions. Specifically, Fact. 3 explained the 12.78% of variance, Fact. 4 the 11.88%, Fact. 5 the 11.00%, Fact. 6 the 10.13%, and Fact. 7 the 9.47%.

**Figure 3:**
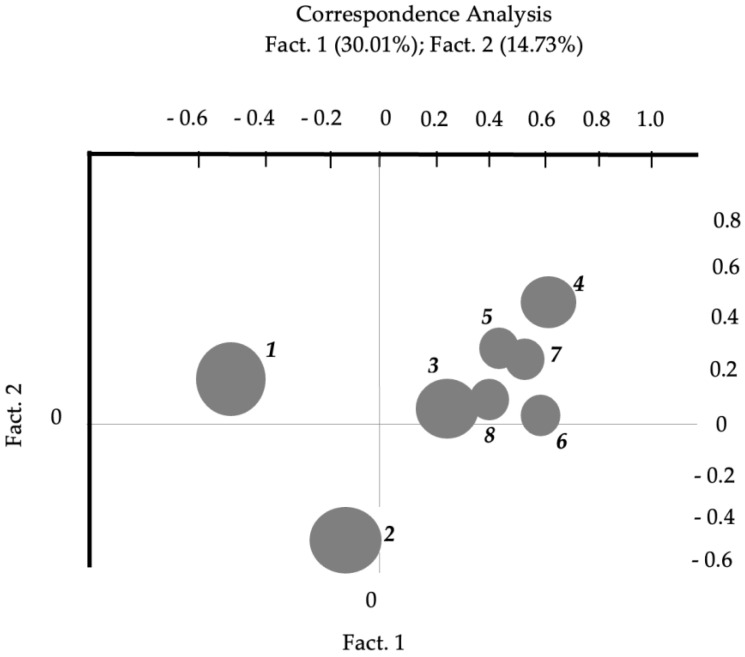
Correspondence Analysis: comparison between the eight group sessions of the intervention.

Figure 3 shows the positioning of each session with respect to a spatial dimension (i.e., axis) set up by a latent dimension (factor) extracted using this type of analysis, which can be referred to as a factorial analysis procedure. In the graph derived from Correspondence Analysis, the vertical axis (Factor 1) comprises session two (in the bottom part of the graph), four, five, and seven (in the top part of the graph). We studied the test value and the associated p-value of each extracted word, considering that higher significant values characterized more important elements in saturating the factorial poles. Then, we considered the semantic difference between the opposed words. The words characterizing Factor 1 reflect a process of “cognitive restructuring” by opposing words related to the illness condition as a deadlock (e.g., “to hospitalize”, “ambulance”, “doctor”, “intensive care unit”, “to die”, and “heavy”) to words related to the group as a supportive context (e.g., “to think”, “work”, “help”, “strategy”, “to cope”, and “relation”). The horizontal axis (Factor 2) comprises session one (on the left) vs. sessions three to eight (on the right). Factor 2 is characterized by words related to physical symptoms (e.g., “pain”, “cold”, “cramp”, “body”, “tachycardia”, and “medicine”) vs. words related to feelings and increased awareness (e.g., “pleasure”, “alone”, “need”, “sadness”, “to relax”, and “to feel”). This factor reflects a switch of focus from the organic to the emotional aspects of the disease, shared and elaborated during the group sessions; in other words, a process of “emotional elaboration”. Table 4 presents test values (saturation values) for lexical units in negative and positive poles of each factor.

## 5. Discussion

This explorative study sought to examine the lived experience of patients previously hospitalized due to COVID-19 infection, considering their verbalizations during a psycho-educational group intervention. Through a qualitative approach and a sematic-pragmatic computer-assisted discourse analysis, we explored in-depth participants’ representations, giving particular attention to the “emotional meaning” process related to their lived experience, and the way they differently narrated and shared feelings within the group during the sessions.

Participants of our study were adults aged from 54 to 80 years old, previously hospitalized due to COVID-19, and then discharged after receiving different therapies based on their health condition after the infection. Therefore, everyone experienced a prolonged period of hospitalization at the clinic where the research took place, along with isolation. According to recent literature on COVID-19 [52,53], patients report a strong sense of loneliness and helplessness caused by isolation (and in some cases, hospitalization), exacerbated by missing their families and close friends. During the post-illness period, these previous feelings could have subsequent repercussions on the general psychosocial well-being, and could persist even several months after recovery, demonstrating long-lasting psychological impact of the COVID-19 disease, and the need for prompt psychological interventions [24,25,26,54,55]. 

In our intervention, participants constituted a group of peers by medical condition (previous infection and then hospitalization for COVID-19 in the same context), in which people had the opportunity to share stories, life experiences, and improve adapting skills. According to literature, the expression of feelings and personal narration within a group of peers conveys processes of identification and mutual recognition in the experience of the other members. In this way, participants realize they are not alone dealing with the consequences of COVID-19, despite the variability in people’s stories. Precisely, if the environment is perceived as safe (e.g., favored by the presence of psychologists as conductors), during the communicative exchange with peers, the person can find new tools and new ways to cope with the situation, to grow and change, overcoming critical issues in an adaptive way. The group setting also promotes empathizing (i.e., literally “putting yourself in the shoes of the other members”), mentalizing (i.e., being able to take the perspective of the other), and reconsidering the events by attributing a new emotional meaning to them [56,57]. 

The first finding of our study was obtained from thematic analysis of elementary contexts which extracted three core clusters, meaning that there were three significant thematic areas in the transcripts of the participants’ group sessions. The narratives of the participants were mostly characterized by descriptions of factual and concrete vicissitudes, a sort of chronicle of events from the first symptom to hospitalization. The cluster explaining the greatest percentage of variance was the one entitled “Disease”, with respect to expressing personal emotional experience related to the disease (i.e., “COVID-19 emotional experience” cluster), or more positive feelings and future orientations (i.e., “Upturn” cluster). However, the analysis showed that during the program, patients’ narrations concerning evolutionary perspective of change, self-confidence, and feeling of mastery of events occur more frequently in the final group sessions (i.e., the seventh and the eighth session), which may indicate a potential positive effect of the psycho-educational group work. 

The relationship between clusters allows us to notice the fluctuation from a condition of helplessness to a future orientation and perception of power over events (or rather, power over how the individual reacts to life events), but also the progressive transition from a factual narration of events to the sharing of an authentic personal story, characterized by increased awareness and a variety of emotions.

Secondly, attention was paid to differences between group sessions (i.e., scheduled modules), and the results allow us to better explore previous findings. At the beginning of this intervention, patients seemed very focused on the concrete management of the disease, showing a difficulty to integrate the event into their own life story and to give it a meaning. Our analysis then revealed that middle and final group sessions were more oriented to cognitive (concerning coping attitude and adaptations skills) and emotional elaboration. 

These preliminary findings seem to be consistent with the extant scientific literature on psychological interventions during the pandemic that highlighted positive effects of psycho-education and CBT techniques on the prompt management of mental health issues [30,34,36]. The psycho-educational structure of the intervention, based on an active “*learner-centered process*” in face-to-face modality at the hospital, appeared as an optimal safe place where participants could rediscover the importance of contact and social support, know and recognize their own difficulties, learn new skills to increase their quality of life and cope with adversities, and finally, know how to use these new skills to improve their everyday life. Furthermore, this is supported by the fact that the learning of new skills by the participants appeared to develop consistently with the progress of the weekly sessions. 

## 6. Conclusions

Findings suggest that there is a congruence between the aims of the group psycho-educational intervention and the experience of the participants during the sessions, compared with the proposed themes. Our analyses revealed an evolution in the discursive style from session one to eight: from an initial “passive” and concrete perspective (i.e., exclusive focus on the disease), to a greater cognitive and emotional elaboration, and to increased participants’ awareness as active agents in the process.

However, we acknowledge several limitations in our study. Firstly, it is a preliminary study where priority was given to the inclusion of the design variable "group sessions". Thus, we did not examine other socio-demographic (e.g., gender and age) or medical variables (e.g., hospitalization duration and prescribed therapy for COVID-19 infection), which could provide more specific insights into the participants’ experiences. 

Secondly, our analyses indicated that certain group sessions were more pertinent and distinct, such as sessions one (“The pandemic and the disease: subjective experience”), session two (“Post-COVID-19 and related symptoms”), and session four (“Nourishing behaviors”), while others were more repetitive with overlapping themes. Further studies will be beneficial to determine whether the intervention program should be modified to minimize redundancy, potentially by reducing the number of sessions, or to find if overlapping themes and sessions are a natural part of the overall elaboration process.

Thirdly, this qualitative study involved a small sample size, and thus, the findings cannot be generalized to larger populations. Therefore, the effectiveness of this intervention, which was beyond the scope of our research, should be examined using larger samples and quantitative methodologies. As planned and required by the study protocol, further research should employ a comparison of psychometric data over time, assessing participants’ psychological health condition before (T0: baseline) and after (T1: end of group sessions) the intervention, as well as during the follow-up, to evaluate the effectiveness of the intervention.

Despite these limitations, we believe that encouraging conclusions can be drawn from this study. In fact, our results suggested that brief psycho-educational group interventions using CBT working instruments and a mindfulness approach could help patients with COVID-19-related mental health issues return to a relatively normal life. In this regard, our study presents a new intervention grounded on classical models and widely spread psychological techniques. Since we are still evaluating its usefulness, effectiveness, and applicability, thus far this intervention has not been applied to other populations. However, preliminary results support the idea of a positive effect on the psycho-physical health of participants. Future investigations may contribute to the definition of an intervention model that could be extended to other populations and contexts. 

One of the positive aspects of the T-LAB software is that it offers standardized procedures of data analysis, which reduces the levels of inference of the researcher, with several advantages in terms of data management. This is important, especially if one considers the current post-pandemic mental health issues [13]; validating targeted interventions is important and timely to support those who suffer from long-term post-COVID-19 psycho-physical symptoms, as well as to assist healthcare professionals in providing them with better and prompt support. 

## Figures and Tables

**Table 1:** Description of the group psycho-educational intervention: main themes, targets, and working instruments for each session.

Nr	Themes	Targets	Working Instruments
1	The pandemic and the disease: subjective experience.	History of illness experience and shared representation. Assessment of baseline psychological well-being using psychometric scales.	Narration of the illness experience in the group: patients tell their own illness story in a safe context. The homework includes a graphic exercise in which patients are asked to indicate physical symptoms on a drawing of the human body. They have a card with a printed stylized human figure, on which participants must mark the part of their body perceived as the most affected by post-COVID-19 symptoms.
2	Post-COVID-19 and related symptoms.	Indicate physical symptoms, recognize psychological impact of the disease in the present; favor a realistic interpretation of the emotional states connected to the symptoms, and recognize the cognitive biases in the elaboration of one’s own experience of illness.	Sharing within the group the homework assigned at the end of session one. Explanation of the Cognitive Model Antecedent Beliefs and Consequences (ABC) [43]: patients are invited to share and discuss the emotional impact of physical symptoms using the ABC scheme. Then, participants receive the ABC scheme and the homework entails using the scheme to analyze the situations experienced during the following week.
3	Dominant emotions and social perception.	Recognize the prevailing emotions in the experience of illness; stigma, isolation, emotional regulation, use of social support.	Sharing within the group the homework assigned at the end of session two: patients share emotions acknowledged through the ABC scheme. The ABC scheme is modified by adding a social resource mapping. The homework entails using the modified ABC scheme to analyze concrete situations during the following week, also evaluating the presence of social support as a resource.
4	“Nourishing behaviors”.	Identify and practice wellness behaviors in everyday life.	Introduction to grounding practices [46]: simple practices are proposed in session four and subsequently included at the beginning of each module. Sharing within the group the homework assigned at the end of session three. Presentation of “Wellness Strategies”, a Mindfulness Based Cognitive Therapy (MBCT) protocol [45,47]. Homework requires that participants identify and introduce well-being behaviors (i.e., “nourishing behaviors”) and grounding practices in their daily lives.
5	Cognitive restructuring.	Recognize and analyze negative automatic thoughts; identify alternative thoughts.	Grounding practices. Sharing within the group the homework assigned at the end of session four. One of the psychologists conducts a psycho-educational session on dysfunctional automatic thoughts. The homework entails using a form to examine automatic thoughts [44]: patients receive a list of possible automatic thoughts, and they must use it to analyze concrete situations, and to recognize their automatic thoughts.
6	Coping strategies.	Use functional coping strategies to promote adaptation.	Grounding practices. Sharing within the group the homework assigned at the end of session five. Psycho-education on adaptive coping strategies. Homework: patients receive information material on coping strategies [44], and a list of possible coping strategies. They must reflect upon which strategies they use the most, and they write them in a diary.
7	Planning and future orientation.	Validate resources; identify concrete goals to pursue in the near future; complete an individual and collective synthesis of group work proposing a representative image, or a metaphor.	Grounding practices. Sharing within the group the homework assigned at the end of session six. Using a form to identify realistic goals [48] through which patients are required to imagine and set realistic goals to be achieved in the near future. Choice of a metaphorical image [49] for the group: patients select an image (among the proposed ones) that allows them to re-read their path through illness using a metaphor. Choice of a shared name of the group. The module ends with the delivery of a certificate of participation.
8	Follow-up.	Verification of objectives, resumption of adaptive strategies, and assessment of current (end of group intervention) psychological well-being with psychometric scales.	Patients talk about the difficulties that emerged since the seventh session, and the strategies used to deal with them.

**Table 2:** General characteristics of the sample (*n* = 18).

	M ± SD (Min; Max)
Age	62.68 ± 8.19 (54; 80)
Days of hospitalization	25.28 ± 22.89 (6; 84)
	***n***
Gender Female Male	10 8
Work Status Full-time job Retired	11 7
Prescribed therapy for COVID-19 infection Pharmacological treatment CPAP Hospitalization in intensive care unit	10 5 3
Mourning of a beloved one due to COVID-19 Yes No	3 15

**Table 3:** Description of clusters in order of explained variance.

Cluster	Explained Variance	Theme	Lexical Units	χ^2^ *	Quotes
Cluster 2: Disease	48.40%	Concrete elements and contextual factors related to COVID-19 disease	Doctor	53.554	“At that moment… the pain I felt at the hospital was just indescribable.” “Due to COVID-19 I was hospitalized urgently and I was like that for a week, dressed the same way… I had nothing, I missed everything. Imagine… I spent the day lying down in bed, I felt pain everywhere and I was alone.”
			COVID-19	44.815	
			Hospital	28.634	
			To hospitalize	16.051	
			To miss	10.972	
			Pain	10.721	
Cluster 3: COVID-19 Emotional Experience	26.45%	Emotional factors related to COVID-19 and inability to move forward	Anxiety	200.804	“It was a nightmare that led to seeing and thinking only of the worst, of the most terrible things... anxiety took over me.” “I tried in every way not to have fear, but I was discouraged just thinking about what was happening.”
			To think	169.876	
			Fear	27.002	
			Nightmare	12.813	
			Anger	11.812	
Cluster 1: Upturn	25.15%	Prospective of positive change	Power	297.442	“My past has deeply affected me, but I try to harness my strength and think that we must always move on, whatever happens in life.” “I felt the power to take back what is beautiful in life, after so much effort. So, this period that I’m leaving behind has been like climbing a mountain [...] After a long time I want take up again everything I never even imagined before.”
			Strength	30.576	
			Life	26.934	
			To move on	12.505	
			To take up again	11.985	
			Motivation	11.664	

* *p* < 0.001.

**Table 4:** Correspondence Analysis: Factors.

*Factor 1: Cognitive Restructuring*				*Factor 2: Emotional Elaboration*			
Negative Pole (Bottom of the Graph)	Test Value *	Positive Pole (Top of the Graph)	Test Value *	Negative Pole (Left of the Graph)	Test Value *	Positive Pole (Right of the Graph)	Test Value *
To hospitalize	−8.5523	To think	5.4622	Pain	−6.4075	Pleasure	3.1728
Ambulance	−7.6656	Work	5.1991	Cold	−6.0669	Alone	2.6933
Doctor	−7.5842	Help	4.3638	Cramp	−4.896	Need	2.1196
Intensive care unit	−7.4841	Strategy	3.5306	Body	−4.2272	Sadness	2.1158
To die	−5.0759	To cope	3.3999	Tachycardia	−3.7914	To relax	2.0202
Heavy	−2.1507	Relation	2.0437	Medicine	−2.2747	To feel	2.0094

* Accepted test values (saturation values) ≥ |1.96| with *p* < 0.05.

## Data Availability

The datasets presented in this article are not readily available because there are privacy and ethical restrictions. Requests to access the datasets should be directed to Sara Molgora, Department of Psychology, Università Cattolica del Sacro Cuore, 20123 Milan, Italy, e-mail: sara.molgora@unicatt.it.
